# Exoscopic supraorbital approach for a suprasellar craniopharyngioma

**DOI:** 10.3171/2023.10.FOCVID23140

**Published:** 2024-01-01

**Authors:** Arpan A. Patel, Erion Júnior de Andrade, Shaarada Srivatsa, Pablo F. Recinos

**Affiliations:** 1 Department of Neurological Surgery, Cleveland Clinic Lerner College of Medicine, Case Western Reserve University, Cleveland, Ohio; and; 2 Section of Skull Base Surgery, Rose Ella Burkhardt Brain Tumor & Neuro-Oncology Center, Neurological Institute, Cleveland Clinic, Cleveland, Ohio

**Keywords:** microscope, endoscopic endonasal, third ventricle, minimally invasive, pterional, optic nerve

## Abstract

The authors present an operative video of a supraorbital craniotomy for resection of a suprasellar, supradiaphragmatic craniopharyngioma. The patient is a 62-year-old female who presented with 3 months of blurry vision secondary to a 2.5-cm suprasellar mass causing compression on the optic nerve. Supraorbital craniotomy was selected due to the supradiaphragmatic location of the tumor and the subsequent disadvantages, including CSF leakage, of other approaches such as the endoscopic endonasal approach. The operative video emphasizes optimizing operating room (OR) setup to improve surgeon ergonomics and comfort. The patient underwent an uncomplicated gross-total resection with subsequent discharge home the day after surgery.

The video can be found here: https://stream.cadmore.media/r10.3171/2023.10.FOCVID23140

## Transcript

This is an operative video describing an exoscopic supraorbital approach for a preinfundibular, supradiaphragmatic craniopharyngioma.

### 0:30 Case Presentation.

The patient is a very pleasant 62-year-old female who presented to clinic with 3 months of partially blurred central and upper temporal quadrant vision in the left eye. The patient was initially self-referred to ophthalmology and was recommended for an MRI brain, which revealed a 2.5-cm enhancing suprasellar, supradiaphragmatic mass compressing the left optic nerve.

### 0:53 Neurological Exam.

On neurological exam, patient had 20/25 vision in her right eye and only finger counting at approximately 8 feet in her left eye. Extraocular movements were intact. The patient had no abnormalities in her pituitary hormone profile.

### 1:07 Preoperative Imaging.

The patient was subsequently referred to neurosurgery. While the MRI was critical to evaluate the lesion characteristics and compression on the optic nerve, a CTA was still needed to evaluate surrounding anatomy including the frontal sinus, the nearby internal cerebral arteries, and to complete our evaluation on the feasibility of potential surgical approaches.

### 1:26 Preoperative MRI.

In this T1 postcontrast MRI, a partially enhancing, cystic suprasellar, supradiaphragmatic lesion is visible, causing compression on the left optic nerve. The left optic nerve is superiorly displaced. The lesion appears to be isolated to the supradiaphragmatic space without extension into the sella below. On sagittal and axial MRI, the lesion is noted to be immediately inferior to the optic chiasm, displacing the nerve superiorly.

### 1:51 Preoperative CT Angiogram.

The CT angiogram reveals no abnormal vasculature or aneurysms and a frontal sinus that’s within normal limits.

### 1:59 Surgical Rationale.

Given the location of this cystic and partially enhancing lesion, craniopharyngioma was highest on the differential diagnosis. Also possible, but less likely, would be a Rathke’s cleft cyst or arachnoid cyst. Given the patient’s progressive decline in visual acuity due to optic nerve compression and unknown pathology of the growing mass, the patient was offered surgery for resection.^1^

### 2:20 Surgical Approaches.

Various surgical approaches are possible to the suprasellar region, including pterional, endoscopic endonasal, and supraorbital. A right-sided supraorbital approach was favored due to lower risk of CSF leak and faster recovery time. The supradiaphragmatic location of the lesion further favored a supraorbital approach due to the high-flow CSF leak that would have been encountered with an endoscopic endonasal approach, which would have required passing through the diaphragm to reach the lesion.

### 2:46 Cadaveric Anatomical Dissection.

A cadaveric dissection of the suprasellar region is shown here to display the anatomical relationship of the infundibulum, optic chiasm, and ICA. The anatomical structures are labeled.^2,3^

### 2:58 Operating Room Setup.

Thoughtful setup of the operating room is integral to success with any surgery; however, this is especially true for surgeries utilizing an exoscope. In addition to the usual considerations for OR setup, surgeries utilizing an exoscope require strategic placement of the 4K monitor in relation to the location and direction of the operating surgeon. The location of the monitor should be in a place that allows the surgeon and the surgical scrub to work at various angles while also being able to easily see the monitor. Multiple monitors can be placed throughout the OR to facilitate this. This graphic represents our room setup for the supraorbital craniotomy. The 4K monitor was placed at the foot of the bed, while the surgical chair was at the head of the bed facing directly toward the monitor. The exoscope base was located to the left of the surgeon with the arm extending above the surgeon’s head. The overall setup of the room and relationship of the surgeon to the 4K monitor facilitates a heads-up position with neutral and comfortable neck positioning for the surgeon. Comfortable positioning for the surgeon allows ergonomic utilization of surgical instruments and stamina for longer surgeries.

### 4:02 Surgical Positioning.

The patient was positioned in the neutral position with the head slightly turned to the left to facilitate the right-sided surgical approach. Here the proposed craniotomy is marked out in order to plan the intra-eyebrow incision. The navigation system is registered and used preoperatively to identify the location of the frontal sinus.

### 4:20 Intra-Eyebrow Incision.

Lidocaine with epinephrine is injected in the dermis. Incision is made inside the right eyebrow through the dermis. Dissection is carried out down to the frontal bone without the use of monopolar electrocautery. Hooks are placed for retraction.

### 4:33 Supraorbital Craniotomy.

The proposed site of the craniotomy is verified with navigation to ensure it is lateral to the frontal sinus. Multiple options exist for creating a supraorbital craniotomy including a high-speed burr versus an ultrasonic burr. The advantages of the ultrasonic burr with the knife attachment include performing a craniotomy without the use of a burr hole as well as a thin footprint of the craniotomy edges, which allow for excellent cosmetic appearance once the bone is replaced.

The height and width of the craniotomy will influence the surgical corridor. In this case, the craniotomy was approximately 1 cm in height and 2.2 cm in width.

### 5:08 Drilling Floor of Anterior Fossa.

Once the craniotomy was performed, the floor of the anterior fossa was drilled to improve visualization of the suprasellar region and the working angle toward the floor. This was easily accomplished with the ultrasonic burr as it is gentle on the surrounding soft tissue, which in this case is the dura. The angle at which the work is performed requires the surgeon to lower the exoscope and point it at an acute angle to visualize toward the floor of the anterior fossa. With the exoscope, this is accomplished easily without multiple further adjustments such as eyepiece and surgical chair.

### 5:42 Dural Opening.

A C-shaped dural opening is performed and the dura is reflected, exposing the frontal pole. Subfrontal dissection is carried down toward the basal cisterns to drain CSF and allow gravity to retract the frontal lobe. A "slider" cottonoid is used here to facilitate retraction while protecting the frontal lobe.^4^ The long focal length capacity of the exoscope allows the scope to remain at distance from the patient which, in turn, allows the surgeon to operate unencumbered. Here, the focal length is set to 51 cm.

### 6:12 Tumor Resection.

Upon visualization of the suprasellar space, a cystic yellow-appearing lesion originating from the pituitary stalk was readily visible. The right side of the tumor is seen making contact with the left optic nerve, which is labeled in yellow. This is consistent with preoperative imaging findings. Careful manipulation of the tumor with microinstruments allowed for the tumor to be dissected free of surrounding tissue, including the ICA, which is seen on the left, labeled in red. Frequent changes in working angles as shown here highlight the advantage of the exoscope over the microscope as the surgeon is able to change the optical viewing angle and then quickly resume operating in the same neutral body and neck position. This is because the surgeon’s sitting and working angle is not coupled to the angle of the scope, as is the case in a traditional surgical microscope.

### 7:02 Tumor Removal.

Once the tumor was dissected free from surrounding structures, its final attachment point was noted to the pituitary stalk. At this point, decision was made to sharply dissect the tumor from the stalk.^5^ The structures are marked, the optic nerve is labeled in yellow, pituitary stalk in blue, and the diaphragm in green.

### 7:21 Irrigation and Hemostasis.

Once the tumor was removed, visual inspection of the suprasellar space was carried out to ensure no damage to surrounding structures and adequate hemostasis.

### 7:31 Dural Closure.

The dura was subsequently reapproximated. Bone flap was replaced with low-profile plating system and bone edges further augmented with application of calcium phosphate bone cement.

### 7:44 Skin Closure.

The incision was subsequently closed with 5-0 absorbable monofilament suture in a subcuticular fashion.

### 7:50 Postoperative Course.

The patient was noted to have a right frontalis palsy that improved at 6-week follow-up. She did not have transient DI and was discharged on postoperative day 1. Pathology report was consistent with craniopharyngioma.

### 8:02 Postoperative MRI.

The postoperative MRI reveals a gross-total resection. Although the left optic nerve is still superiorly displaced, the nerve is thoroughly decompressed.

### 8:11 Discussion.

The tools available to neurosurgeons for visualization have continued to increase in number and complexity over the years. Advancements in optical technology have resulted in a wide range of tools, including microscopes, endoscopes, and now exoscopes. The OR setup for each one of these entities is unique to one another, and understanding those differences lays the groundwork for success with the surgery. Overall, we have found that the exoscope allows for better teaching of trainees, surgeon ergonomics, and improves surgical flow, especially in surgeries with challenging visualization angles.

## Disclosures

Dr. Recinos reported personal fees from Stryker and stock ownership in Acera Surgical Inc. outside the submitted work.

## Author Contributions

Primary surgeon: Recinos. Assistant surgeon: Patel, Júnior de Andrade. Editing and drafting the video and abstract: all authors. Critically revising the work: Recinos, Patel, Srivatsa. Reviewed submitted version of the work: Recinos, Patel, Srivatsa. Approved the final version of the work on behalf of all authors: Recinos. Supervision: Recinos.

## Supplemental Information

### Patient Informed Consent

The necessary patient informed consent was obtained in this study.

## References

[1] Prieto R Pascual JM Barrios L Optic chiasm distortions caused by craniopharyngiomas: clinical and magnetic resonance imaging correlation and influence on visual outcome World Neurosurg 2015 83 4 500 529 25308925 10.1016/j.wneu.2014.10.002

[2] Almeida JP de Andrade E Reghin-Neto M Radovanovic I Recinos PF Kshettry VR From above and below: the microsurgical anatomy of endoscopic endonasal and transcranial microsurgical approaches to the parasellar region World Neurosurg 2022 159 e139 e160 10.1016/j.wneu.2021.12.023 34906753

[3] Almeida JP Junior de Andrade E Vescan A Surgical anatomy and technical nuances of the endoscopic endonasal approach to the anterior cranial fossa J Neurosurg Sci 2021 65 2 103 117 33245220 10.23736/S0390-5616.20.05086-9

[4] Shao J Borghei-Razavi H Kshettry VR Lim M Recinos PF Cottonoid sliders: a simple and cost-effective tool for retractorless intracranial surgery Oper Neurosurg (Hagerstown) 2020 19 4 E428 E431 32357243 10.1093/ons/opaa099

[5] Prabhu VC Brown HG The pathogenesis of craniopharyngiomas Childs Nerv Syst 2005 21 8-9 622 627 15965669 10.1007/s00381-005-1190-9

